# The impact of contextualization on immersion in healthcare simulation

**DOI:** 10.1186/s41077-016-0009-y

**Published:** 2016-03-08

**Authors:** Henrik Engström, Magnus Andersson Hagiwara, Per Backlund, Mikael Lebram, Lars Lundberg, Mikael Johannesson, Anders Sterner, Hanna Maurin Söderholm

**Affiliations:** 1 School of Informatics, University of Skövde, Box 408, 541 28 Skövde, Sweden; 2 Centre for Prehospital Research, Faculty of Caring Science, Work Life and Social Welfare, University of Borås, 501 90 Borås, Sweden; 3 Swedish Armed Forces Centre for Defence Medicine, Box 5155, 426 05 Västra Frölunda, Sweden; 4 Centre for Prehospital Research, Swedish School of Library and Information Science, Faculty of Librarianship, Information, Education and IT, University of Borås, 501 90 Borås, Sweden

**Keywords:** Medical simulation, Immersion, Fidelity, Contextualized

## Abstract

**Background:**

The aim of this paper is to explore how contextualization of a healthcare simulation scenarios impacts immersion, by using a novel objective instrument, the Immersion Score Rating Instrument. This instrument consists of 10 triggers that indicate reduced or enhanced immersion among participants in a simulation scenario. Triggers refer to events such as jumps in time or space (sign of reduced immersion) and natural interaction with the manikin (sign of enhanced immersion) and can be used to calculate an immersion score.

**Methods:**

An experiment using a randomized controlled crossover design was conducted to compare immersion between two simulation training conditions for prehospital care: one basic and one contextualized. The Immersion Score Rating Instrument was used to compare the total immersion score for the whole scenario, the immersion score for individual mission phases, and to analyze differences in trigger occurrences. A paired *t* test was used to test for significance.

**Results:**

The comparison shows that the overall immersion score for the simulation was higher in the contextualized condition. The average immersion score was 2.17 (sd = 1.67) in the contextualized condition and −0.77 (sd = 2.01) in the basic condition (*p* < .001). The immersion score was significantly higher in the contextualized condition in five out of six mission phases. Events that might be disruptive for the simulation participants’ immersion, such as interventions of the instructor and illogical jumps in time or space, are present to a higher degree in the basic scenario condition; while events that signal enhanced immersion, such as natural interaction with the manikin, are more frequently observed in the contextualized condition.

**Conclusions:**

The results suggest that contextualization of simulation training with respect to increased equipment and environmental fidelity as well as functional task alignment might affect immersion positively and thus contribute to an improved training experience.

## Background

This work draws on a case study collaboration bringing together expertise from different simulation areas (medical simulation and serious games) with the overall focus to improve simulator training in prehospital emergency care. The prehospital context, i.e., all activities taking place from an initial alarm call until a patient is delivered at the hospital emergency unit, is a complex process [1] that includes many different dimensions and challenges: e.g., communication and teamwork skills, transport and driving, medical skills, and decision-making. Today, current training practice (e.g., in the regional ambulance organizations that participated in our project) is that different aspects are trained in isolation, e.g., medical skills using patient manikins, driving using driving simulators, and teamwork in teamwork sessions. As discussed by Rice [2], many types of skills, e.g., cognitive, social, and non-technical skills are neither immediately challenged nor synthesized in traditional manikin-based simulation, unless time and effort is put into the environment in which the manikin is placed. In cases when the learning goal is to train complex healthcare processes or team collaboration, better learning could potentially be created through increasing both breadth and detail in the simulated scenario. In the context of this research, breadth refers to the number of activities and phases, i.e., the overall process from dispatch to ER handover. Detail refers to increasing the realism and enriching each phase of that process so that it better mirrors the whole array of activities in terms of interactions, skills, tools, and information (audio, tactile, interactive, communicative, technical, etc.) that prehospital nurses carry out and use during an ambulance mission. The idea of having increased richness in terms of covering the whole prehospital chain is in line with the 1990s refinement of aviation simulators when context was taken into account by using full missions. According to, e.g., Rudolph et al. [3] and Dieckmann et al. [4], this was one of the factors that led to a dramatically improved aviation safety. Similarly, we believe that taking mission context into account, e.g., by including the driving route activity from the ambulance station and back and recreating the interior characteristics of a patient’s home, as well as present changes in the emotional and physical state of the patient, is likely to increase the physical, conceptual, and emotional realism [3] of the simulation.

In this work, we assess a highly contextualized mixed-reality approach to simulation design. This approach strives to increase immersion through a combination of role-play, technical props, and contextualization of work tasks. In healthcare education, role-play has been used for over 40 years varying from dialogue between students portraying a nurse and a patient to more complex situations [5]. In simulation, role-play can be used as a way to integrate the communication processes that normally are present in real care situations [6]. When designing our simulation approach, we strived to create environments, activities, and scenarios that included natural role-play, e.g., interaction between the trainees (prehospital nurses) and the patient, with bystanders or family members, with attending physicians through phone and handover, and within-team collaboration when prehospital nurses are working together. In addition, a combination of physical and digital props was used to create a more engaging environment.

The participants’ involvement in a simulated scenario can be characterized by a number of different terms (Andersson Hagiwara M, Backlund P, Maurin Söderholm H, Lundberg L, Lebram M, Engström H: Measuring participants’ immersion in healthcare simulation: the development of an instrument, Forthcoming), such as flow, presence, cognitive absorption, buy-in, suspension of disbelief, and the as-if concept. This paper studies participants’ involvement in simulator training in terms of immersion [7, 8]. In healthcare simulation, the term immersion is often used in relation to virtual reality (VR) and seen as being determined by technical components. Here, we use a definition more commonly adopted in the game research community, pertaining to immersion as a subjective psychological experience [7–11]: “the subjective impression that one is participating in a comprehensive, realistic experience” [12] (p.66). This definition emphasizes immersion as lived experience rather than a property of a technical environment and thus helps us to conceptualize what and how that might bring prehospital personnel engaged in a training scenario into and out of an immersive state. One important property of immersion, which differentiates it from, e.g., flow, is that it is a continuum (from engagement to total immersion). This makes it meaningful to quantify the level of immersion. The primary approach today to measure immersion is through questionnaires, e.g., Jennett et al. [8], capturing participants’ experience post taking part in an activity. So far it does not, to our knowledge, exist in any measure that can be used to observe immersion in a non-intrusive, objective way. In another paper, we present the development and validation process of the Immersion Score Rating Instrument (ISRI) designed to observe and measure immersion in mixed-reality healthcare simulation. This instrument consists of 10 triggers that indicate reduced or enhanced immersion among participants in a simulation scenario. Triggers refer to events such as jumps in time or space (sign of reduced immersion) and natural interaction with the manikin (sign of enhanced immersion) and can be used to calculate an immersion score.

In the study presented in this paper, we have manipulated breadth and detail in two scenarios to explore how this might affect participants’ immersion (see Table 1 for an overview). Thus, the research question addressed in this paper is: How is participants’ immersion during a simulated scenario affected by contextualization? In order to investigate this, we apply the newly developed ISRI, which allows us to (1) observe and measure immersion at a general level, (2) identify variations during different phases of a healthcare scenario, and (3) analyze individual triggers that might reduce or enhance participants’ immersion.

**Table 1:** Experiment condition design per ambulance mission phase

Mission phase	Basic	Contextualized	Fidelity adjustments
(1) Dispatch, ambulance	Dispatch delivered from the instructor during introduction	Through a realistic two-way communication system	Equipment fidelity, functional task alignment
	Oral information from the instructor	Full visualization, in a driving simulator, of an actual turnout from the station to the address (4 min). Communication with the dispatch center is possible.	Equipment fidelity, environmental fidelity, functional task alignment
(2) On scene assessment	The instructor informs the team that they are at the scene and that they can start working. Equipment is already in place.	Crew physically relocate themselves and equipment to the patients’ apartment.	Functional task alignment
		The team enters an apartment (simulated in a lecture room) with physical props and with interiors projected on the walls—which may indicate, e.g., the lifestyle of the patient or give clues about the situation. Ambient sounds and a dog barking behind a half open door were used to further enrich the environment.	Environmental fidelity
(3) Initial patient assessment	The team interacts with the manikin in a lecture room that represents the home of the patient.	The team interacts with the manikin in the apartment.	Environmental fidelity
(4) On-scene treatment	Medicine is delivered by informing the instructor of the action.	Medicine is delivered in a realistic way using RFID tags.	Equipment fidelity
	Calls to medical control and ECG transmission are handled via the instructor.	Calls to medical control and ECG transmission are handled in a realistic way.	Equipment fidelity, functional task alignment
(5) Scene departure and transport	The manikin is loaded to the stretcher. The team stays in place but verbalize/report to the instructor that they load the patient into the ambulance.	The manikin is loaded to the stretcher, brought out of the apartment, and then loaded into the ambulance/driving simulator.	Equipment fidelity, environmental fidelity, functional task alignment
	Driving is not a part of the simulation but the team may discuss the case and inform the instructor of what they would have done during transport.	The simulated trip to the emergency department takes 7 min during which additional treatment is carried out in the ambulance.	Equipment fidelity, environmental fidelity, functional task alignment
(6) Patient handover to emergency department	The team reports to the attending emergency physician.	The team reports to the attending emergency physician.	

The rightmost column indicates which fidelity dimensions that have been affected by the contextualization

### Fidelity in healthcare simulation

During a simulation, immersion is affected by several different factors (e.g., physical, structural, communicative, personal, or contextual). In healthcare simulation, these are commonly referred to as different categories of fidelity [13] and often discussed in relation to the fidelity level of a manikin. Archer et al. [14] describe fidelity by using three dimensions: equipment fidelity, which concerns how closely the simulator resembles the real system it refers to. The second dimension, environmental fidelity, refers to the context in which the simulator is placed. Finally, the third dimension is called psychological fidelity and refers to the degree to which the trainee perceives or accepts the simulation to be “real”. According to Tun et al. [15], contemporary definitions of fidelity typically refer to the level of realism of a simulation. Furthermore, their review of the fidelity concept reveals that definitions are not clear and may refer to either the physical (engineering) aspects of a simulation, i.e., the extent to which a simulation reflects the physical properties of the real-world concept, or its subjective dimension, called psychological or perceptual fidelity. As fidelity is not a clearly defined concept, it has recently been criticized for being too imprecise [16]. Hamstra et al. even propose to abandon the term fidelity and replace it with the terms physical resemblance and functional task alignment [16]. The benefit of doing so is that it allows us to focus more on the functional alignment with the learning task rather that the current overemphasis of physical resemblance. Accordingly, we classify the first two dimensions proposed by Archer et al. [14] as dimensions of the physical resemblance whereas the third dimension concerns the buy-in to the simulation, i.e., the degree to which the trainee accepts the situation as believable and suitable for its purpose. Functional task alignment is another matter, and Hamstra et al. [16] emphasize the importance of close alignment between the clinical task and the simulation task. Functional task alignment can be strengthened by an appropriate correspondence between the simulator and the applied context. Similar staffing and spatial arrangements can help to achieve this. In the case of prehospital training, we argue that these features may be present in an enriched scenario context for the patient simulator. This does not mean that the physical resemblance of the patient simulator is unimportant, only that it should be considered with respect to the training goal, in our case prehospital care.

As can be seen from the above discussion, fidelity is not a clear concept and neither is its relation to learning. According to Hamstra et al. [16], there is a positive relation between cognitive engagement and learning outcome. However, physical resemblance is only one parameter when enhancing learner engagement. Rettedal [17] discusses participants’ perceptions of realism regarding simulated scenarios and points out the suspension of disbelief as a central concept for successful simulation. Horcik et al. [18] refer to this and claim that involvement in simulation requires that participants suspend disbelief. Based on studies of various immersive interfaces, Huiberts [11] asserts that immersion, in a digital environment, can enhance education by allowing multiple perspectives, situated learning, and transfer. The results from a relatively recent study of the learning outcomes of science educational games by Cheng et al. [19] indicate that that immersion leads to a higher gaming performance, which in turn plays a role in learning performance.

**Fig. 1:**
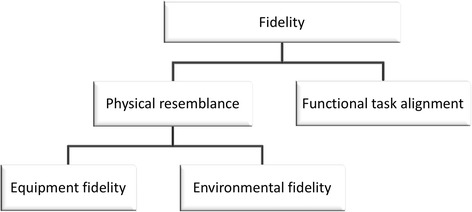
The dimensions of fidelity

To summarize, we see that fidelity is a complex phenomenon which lacks a clear definition. We acknowledge that physical resemblance and functional task alignment are important factors when discussing fidelity and the effectiveness of simulation training. The physical resemblance does not only relate to the manikin but also includes the equipment as well as environmental fidelity. We summarize our view of fidelity in Fig. 1. In our case, this means that we not only utilize a patient simulator manikin as well as physical props to create some sense of realism but we also consider functional task alignment when including tasks from the whole prehospital process to be carried out according to standard procedure.

## Methods

### Study setting

The experiment was conducted in November 2014 as a collaborative multidisciplinary effort between serious games and prehospital care researchers from two universities in Sweden and training officers from a regional ambulance center.

### Participants

Twelve teams (24 professionally active ambulance nurses) from four different healthcare organizations in the surrounding region participated in the study. All participants were working full time as ambulance nurses and had earlier experience from simulation training.

### Ethics

The study was approved by the research ethics adviser at the University of Borås, Sweden and conducted in accordance with the ethical recommendations of the Swedish Research Council [20]. During an introductory session to the experiment, the principal investigator informed study participants about the participation in the study, their rights, and our responsibilities as researchers. Informed oral and written consent was obtained from all participants.

### Study design

The study had a randomized controlled crossover design comparing immersion between two types of simulation training conditions: one basic, mirroring how training currently is done in the regional ambulance organizations participating in the study, and one contextualized, where we strived to capture more of the complexity of the prehospital work process. Table 1 illustrates the design of the two experiment conditions per central phases of an ambulance mission. The phases were determined by factors such as change of physical location (transport) or different segments of the on-scene assessment and treatment where the highest number of, or most important, decisions are made [21]. This resulted in the six phases presented in Table 1. In the contextualized simulation design, we utilized a mixed-reality approach, recreating parts of the environment through physical props, e.g., using a real ambulance as interface to the simulated driving. The same ambulance was also used for actual loading and for patient care during transport to hospital. In both conditions, a Laerdal SimMan 3G simulator was used. The manikin was operated via Wi-Fi where the operator was playing the role of the patient by communicating via the manikin’s integrated speaker system.

### Randomization and control

**Fig. 2:**
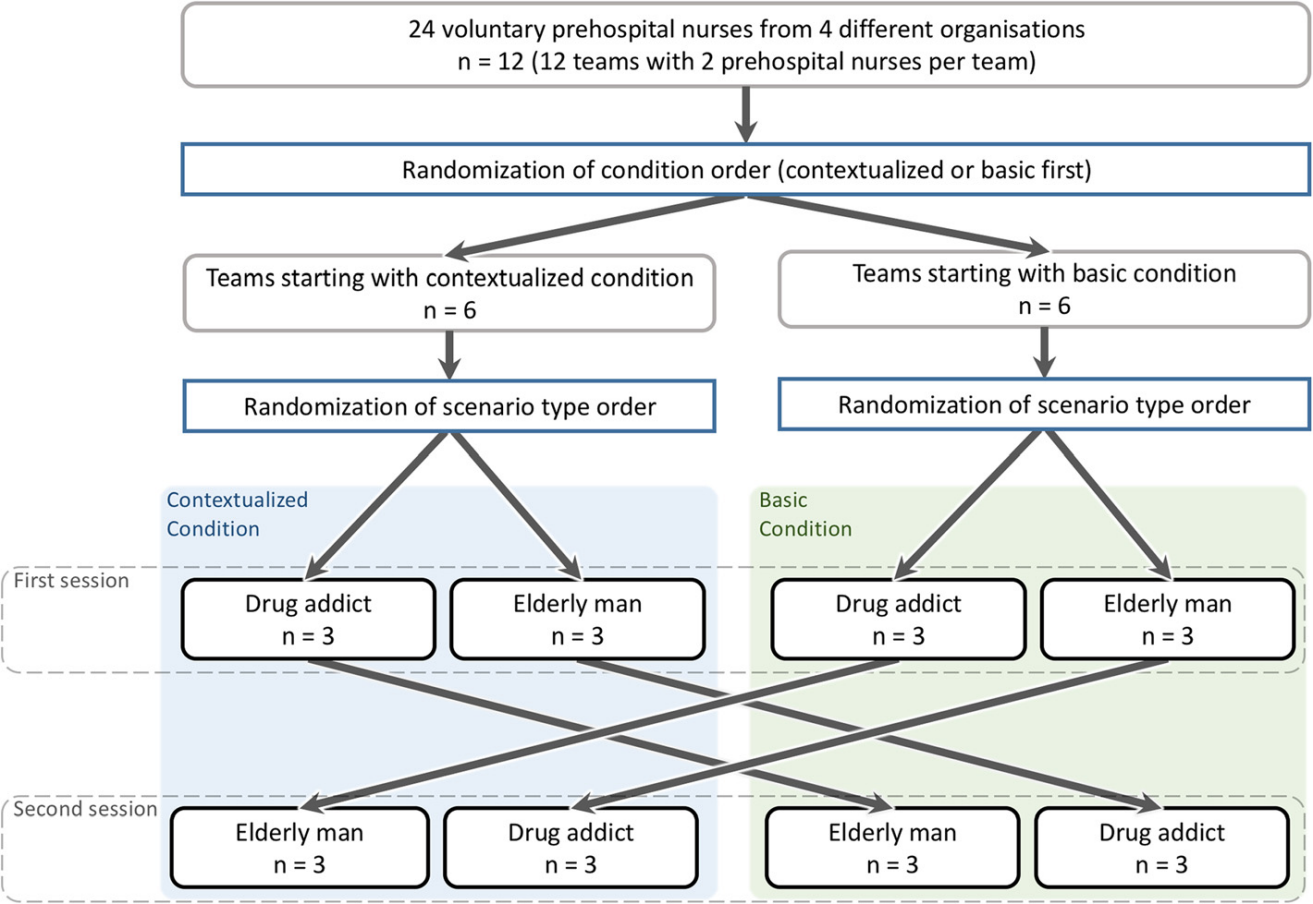
Flowchart, randomized controlled crossover design

In each condition, two different medical scenarios were used. Upon arrival at our facility, participants were randomly assigned to which condition and medical scenario to start with. Hence, the scenarios were organized in blocks in order to vary: (1) the type of medical scenario (“elderly man with respiratory distress” or “drug addict with respiratory distress”) in each of the conditions (contextualized/basic) and (2) the order in which participants did the scenarios (Fig. 2).

### Experiment protocol

When arriving at the ambulance station, participants were given an introduction to the study and its aims, their participation including reading and signing consent forms, and responding to a background information questionnaire. Next, they were subjected to the two different simulation conditions (contextualized/basic). Before each condition, participants were introduced to the simulation by the experiment leader and given time to familiarize themselves with the manikin and any equipment provided. During each condition, participants were working through an ambulance mission as described in Table 1. Each block was concluded by a debriefing session with the attending emergency physician who participated in the handover phase. In all, each team spent 5 h (including lunch and refreshment breaks) at the ambulance facility where the study was carried out.

### Measures

The entirety of all the simulations was recorded by a number of video recorders and one handheld audio recorder. To analyze the video recorded sessions, we utilized a recently developed instrument, ISRI. This instrument consists of 10 triggers (T1-T10) that are used to determine participants’ immersion during the simulation. Here, a trigger refers to an event in the simulation that was considered a sign for reduced or enhanced participant immersion. As illustrated in Table 2, triggers T1-T7 indicate issues that reduce immersion, i.e. breaks in immersion, while triggers T8-T10 indicate enhanced immersion. Details of the ISRI development process including the complete trigger inventory are reported in (Andersson Hagiwara M, Backlund P, Maurin Söderholm H, Lundberg L, Lebram M, Engström H: Measuring participants’ immersion in healthcare simulation: the development of an instrument, Forthcoming).

**Table 2:** Trigger definitions and directions (i.e., if they indicate reduced or enhanced immersion)

Trigger#	Definition	Direction
T1	Destructive interaction between participants and persons outside the scenario	Negative
T2	A participant expresses that the expected equipment is missing or not functioning normally.	Negative
T3	Disturbing jumps in time and/or space	Negative
T4	All or part of the operations are pretended.	Negative
T5	Unnatural interaction with the patient and/or another person in the scenario	Negative
T6	Uncertainty in what is expected or can be done in the simulated scenario	Negative
T7	Technology that would not be part of a natural context disturbs the participants.	Negative
T8	Natural responses to stimuli in the scenario	Positive
T9	Natural interaction with the simulator	Positive
T10	Natural interaction and/or verbal communication with another person in the scenario	Positive

### Data analysis

#### Trigger and timestamp assignment

**Fig. 3:**
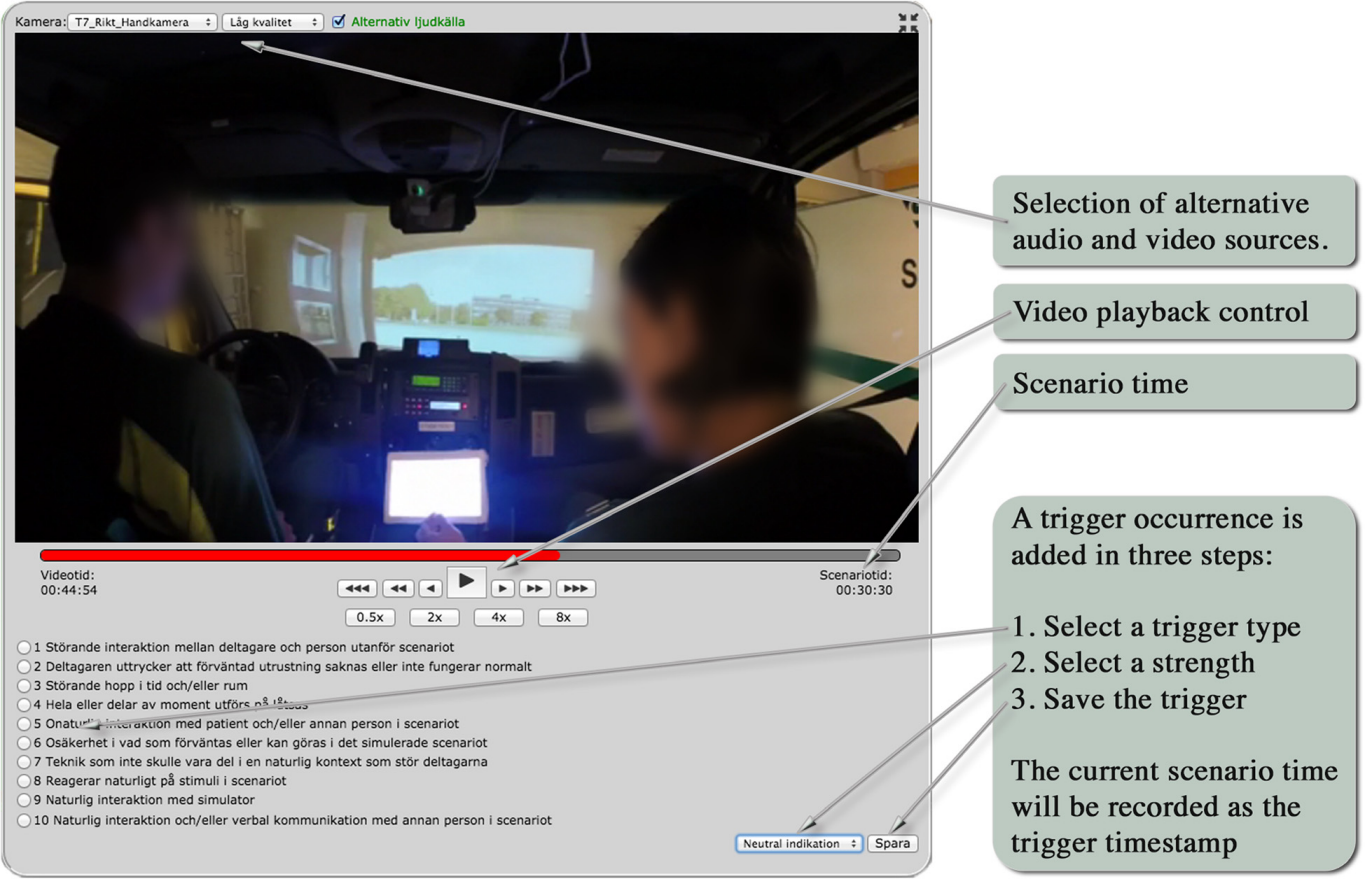
Video analysis interface (showing video from phase 1, ambulance en route)

When applying ISRI to the recorded sessions, five researchers first took part of interrater training and then analyzed two to three teams each (i.e., in total four to six sessions per rater). Here, a rater watched the recording of a session in a computerized system where video and trigger assignment input was integrated (Fig. 3). When a situation arose that indicated reduced or enhanced immersion, the rater stopped the video and selected an appropriate trigger, optionally including a subheading. For each assigned trigger, the rater also indicated the trigger strength from 1 (weak indication) to 3 (strong indication). A two-way mixed, consistency, average-measures intraclass correlation (ICC) of overall interrater reliability showed excellent results (with ICC = 0.92). Next, all video recordings were manually timestamped in time intervals corresponding to the phases defined in Table 1.

#### ISRI score calculation

The triggers assigned during a time interval are used to compute a summarizing ISRI score. During a time interval of ∆ minutes, each trigger *t* (1 ≤ *t* ≤ 10) occurs *n*_*t*_ times. The total number of trigger occurrences during the interval is hence ${\displaystyle {\sum}_{t=1}^{10}}{n}_t$. Each occurrence *i* (1 ≤ *i* ≤ *n*_*t*_) of a trigger *t* has been assigned a strength *ω*_*ti*_ (1 ≤ *ω*_*ti*_ ≤ 3). The total immersion score, *s*, for the interval is computed as

1 $$ s=\frac{{\displaystyle {\sum}_{t=8}^{10}}{\displaystyle {\sum}_{i=1}^{n_t}}{\omega}_{ti}-{\displaystyle {\sum}_{t=1}^7}{\displaystyle {\sum}_{i=1}^{n_t}}{\omega}_{ti}}{\Delta} $$

The immersion score is hence the sum of all strengths assigned to positive triggers (T8–T10) minus the sum of all strengths assigned to negative triggers (T1–T7) divided by the length of the interval in minutes. The scores reported in this paper are computed based on the whole simulation and on its individual phases. The length of these varies depending on teams’ performances and the nature of the condition. By dividing the trigger strength with time, the immersion score can be used to compare sessions and phases with different durations. In this way, the ISRI score can be said to be normalized to provide an intensity value. An alternative would be to use only the sum of strengths, which would result in a metric that would aggregate the trend over time. However, a long running scenario with a positive immersion trend would then get a much higher score than a similar short scenario. This would make it difficult to compare the score from different types of condition durations as, for example, is possible with a post-questionnaire instrument.

#### Comparison between conditions

To determine differences in ISRI score between the two conditions, a paired *t*-test was used.

#### Order effects

Potential order effects were explored by calculating the difference in ISRI score between the contextualized and basic condition. Independent *t* tests on the differences were then conducted with order and type of scenario as independent variables.

### Statistical analysis

All statistical analyses were performed using the statistical software program SPSS 21.0 (SPSS Inc., Chicago, IL).

## Results

All teams worked through the entire simulation in both conditions. On average, the contextualized condition took 34 min (sd = 3.5), ranging from 28 to 39 min, and the basic condition took on average 15 min (sd = 3.4), ranging from 10 to 20 min. This is a reasonable difference since the contextualized condition included more and longer steps, e.g., actual driving mirroring realistic transport times.

### Overall immersion differences between conditions and phases

**Fig. 4:**
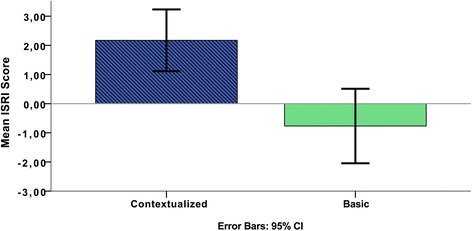
The ISRI score (contextualized vs. basic) for the whole scenario (*n* = 12). The difference is significant at *p* < .001

For all groups, the overall immersion score for the simulation was higher in the contextualized condition. The average immersion score was 2.17 (sd = 1.67) in the contextualized condition and −0.77 (sd = 2.01) in the basic condition (Fig. 4). The difference is significant at *p* < .001, using a paired *t* test.

In all, the overall immersion trigger analysis clearly shows higher immersion in the contextualized condition than in the basic. The assignment of scenarios (“elderly man…” or “drug addict…”) to conditions did not have any effect (*p* = .911) on the difference in ISRI score between conditions. The average difference was 2.89 for teams with the “elderly man…” scenario in the contextualized condition and 2.99 for teams with the “drug addict…” in the contextualized condition. There is a tendency that the order of conditions has an effect on the ISRI score. The average difference was 3.44 for teams starting with the basic condition and 2.44 for teams starting with the contextualized condition. Although this difference is not significant (*p* = .246), it is still notable and should be considered in future studies.

**Fig. 5:**
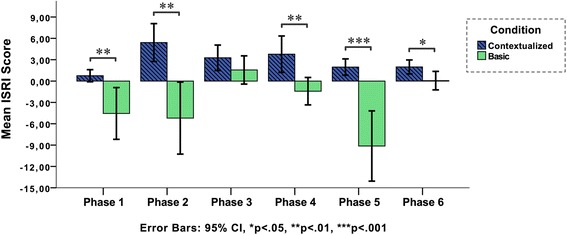
The ISRI score (contextualized vs. basic) for each phase of the scenario (*n* = 12)

These results do not, however, tell us anything about when during the simulation the participants’ immersion is higher or lower. Therefore, we have explored in which of the mission phases (as defined in Table 1) differences in immersion were located. Immersion score within each condition was calculated per each of the phases. Figure 5 illustrates how teams’ immersion varies during the different phases of the simulation.

Here, we can clearly see that the contextualized condition has less variance during the process, and that immersion increases during phase 2 (on scene assessment) and 4 (on scene treatment), and decreases in phases 3 (initial patient assessment), 5 (scene departure and transport), and 6 (patient handover to emergency department). In the basic condition, immersion is more fluctuating, with its relative highest points in phase 3 (initial patient assessment) and lowest in phases 2 (on scene assessment) and 5 (scene departure and transport). As Fig. 5 illustrates, phase 3 is the only positive peak in the basic condition.

The fact that the differences between the conditions are largest in phases 2 and 5 is not surprising. In these phases, the participants in the basic condition had to pretend that they were loading and transporting, while the contextualized condition allowed actual loading and real-time driving in a real ambulance vehicle (integrated with a driving simulator). Immersion differences are smallest in phases 3 and 6, the two phases that were most similar in terms of simulation design and equipment (see Table 1). Hence, in order to understand what the immersion differences consist of, and what fidelity dimensions, activities, or props in our simulation design that might affect these, we need to investigate the trigger distribution per each phase in more detail.

### Understanding immersion differences for different activities and mission phases

**Fig. 6:**
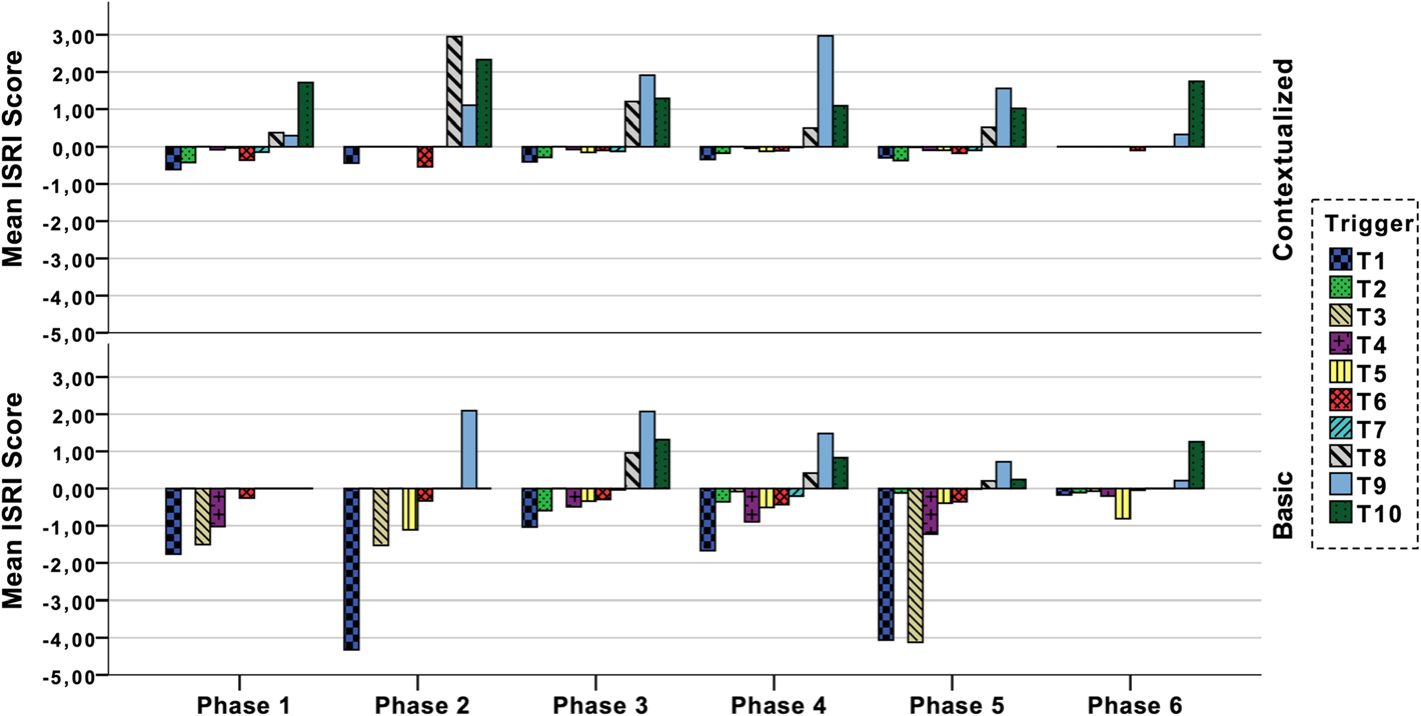
The ISRI score per phase split into the individual triggers. The sum of the 10 trigger values constitutes the immersion score shown in Fig. 5. Each trigger value is the mean of all teams (*n* = 12)

The immersion score is computed from the individual trigger groups, which can contribute negatively or positively to the total score (see Eq. 1). To explore the underlying factors, the scores shown in Fig. 5 have been decomposed into individual trigger group components, shown in Fig. 6. The sum of all trigger groups for a phase in Fig. 6 corresponds with the mean value of the corresponding phase for that condition (shown in Fig. 5). For example, phase 6 in the basic condition has a mix of positive and negative triggers which balance out and result in an immersion score close to zero, as can be seen in Fig. 5. Hence, Fig. 6 helps in visualizing the distribution of the different triggers beyond the computed mean value.

#### Differences in trigger occurrences

The basic condition is dominated by two negative triggers: destructive interactions (T1—typically interventions of the instructor) and jumps in time and/or space (T3). These triggers are dictating the score during phase 2 (on-scene assessment) and phase 5 (scene departure and transport) in the basic condition, as compared to the contextualized where positive triggers are dominating these phases. In fact, jumps in time and/or space (T3) are almost not present in the contextualized condition and destructive interactions (T1) is present on a relatively constant low level during the whole scenario except in the last phase. For the whole simulation, both these differences are significant at *p* < .001, using a paired *t* test.

#### Changes in trigger occurrences

As can be seen in Fig. 5, the basic condition exhibits a shift from a positive overall immersion score during phase 3 (initial patient assessment) to a negative during phase 4 (on-scene treatment). As illustrated in Fig. 6, this shift consists of increased destructive interactions (T1), followed by an almost proportional decrease of natural responses to stimuli (T8), natural interaction with the simulator (T9), and with participants (T10). A natural response in the contextualized condition is, e.g., when the participant comforts the patient manikin by patting its arm during transport to hospital; while an unnatural response could be events such as the participant driving the ambulance lets go of the steering wheel and starts doing something else while being en route (contextualized) or both participants engaging in patient treatment even though they (in the basic condition) have verbalized that they are en route driving to the ER. There is also a clear increase in pretended operations (T4), such as the participant just putting down a heap of ECG cables on the patient’s chest while verbalizing “I’m taking an ECG”.

In contrast, the difference between phase 3 and phase 4 is much less apparent in the contextualized condition where the total immersion score instead increases slightly (Fig. 5). Here, negative triggers (T1–T7) are almost unchanged between phases; instead, there is a change in the distribution of positive triggers. Natural interaction with the simulator (T9) increases while there are fewer natural responses to stimuli in the simulation (T8).

#### Unnatural execution of operations and technological distractions

There is an almost complete absence of pretended operations (T4) in the contextualized condition, while appearing in most phases of the basic condition. The presence of this difference in phases 3 and 4 is somewhat surprising, as the same manikin and technical and medical equipment are used in both conditions, and hence presents similar conditions and tools for patient care activities. These differences are significant at *p* < .05, using a paired *t* test. Interestingly, technological distractions (T7) is the least occurring trigger, it is barely present in any of the conditions or phases.

### Differences in similarly designed phases

The three phases initial patient assessment, on-scene treatment, and patient handover to ER (phases 3, 4, and 6) are most similar between the conditions in terms of how the simulation was designed. As shown in Fig. 5, this similarity is reflected as small differences in total immersion. Now we turn to what this looks like in terms of individual triggers. The mix of triggers during phase 3 (initial patient assessment) is similar in the contextualized and basic conditions. The dominating trigger in both conditions is natural interaction with the simulator (T9), which here is necessary to do in order to determine a diagnosis. After this phase, T9 is gradually decreasing in the basic condition. Instances of unnatural interaction with manikin or participants (T5) are however clearly more present in the basic condition. For the whole simulation, the difference in T5 occurrences is significant at *p* < .05, using a paired *t* test.

## Discussion

Overall, our analysis shows higher immersion in the contextualized condition than in the basic. The overall immersion score is higher and participants’ immersion does not fluctuate as much during the different phases as it does during the basic scenario. Triggers pertaining to events that might be disruptive for participants’ immersion, such as interventions of the instructor and illogical jumps in time/space are less frequent in the contextualized condition, while triggers referring to natural interaction with the manikin or other participants are more frequent. Furthermore, operations, tasks, and interactions are to a higher extent conducted in a natural, more realistic way in the contextualized condition. This suggests that contextualization might support a better workflow during a simulation scenario, provide less interruptions, reduce uncertainty of what to do next, and that it promotes natural execution of tasks, as well as natural interaction with manikin or participants. Although the overall immersion is higher in the contextualized condition, both conditions have a mixture of triggers that enhance or reduce immersion. This resembles the results reported in [18] where participants’ concerns shifted between issues related to the targeted work and issues related to the simulation. They state that “a unique and stable immersion was never observed” [18] (p.98).

Contextualization includes however a higher number of technological components to increase environmental fidelity and functional task alignment, e.g., identical functional replicas of the same IT and telecommunication equipment normally used by prehospital nurses. Even so, there were minimal occurrences of technological distractions (T7) in both conditions. Hence, it appears that additional technical components did not introduce additional distractions.

Phases 3, 4, and 6 were the ones most similar between conditions in terms of fidelity. Although differences in total immersion between the conditions are smallest in these phases, there are some interesting differences in trigger occurrences. In the basic condition, for example, the natural interaction with the simulator is declining after the initial patient assessment. This is in contrast to the contextualized condition where it increases. Hence, even though the equipment fidelity (manikin, medical equipment, and tools) was close to the same in these phases, it appears that the increased environmental fidelity (dog barking, worried neighbors present) and functional task alignment (interactions with medical control, dispatch info, patient data) might compensate for potential frustration or immersion disruptions induced by the manikin. The increase of T9 (natural interaction with the simulator, here by, e.g., physically touching, talking calmly to, and comforting the patient) in phase 4 may indicate that the participants at this point in the simulation perceive the manikin as a real patient who needs to be involved when treatment is given. This suggests that role-playing plays a crucial role in our results, bringing a positive enforcement of natural interaction with the manikin and within the team as well as with other participants in the simulation. This resonates with the suggestions by Dieckmann et al. [4], that different types of fidelity can influence immersion in different ways. For example, it is probably possible to reach a high level of immersion without a high level of physical fidelity; instead functional task alignment where a realistic sense of stress or time pressure is created, or natural compassionate interaction with a distressed patient, influence immersion positively.

According to the findings by Dieckmann et al. [22] of perceived realism in healthcare simulation, it is the interaction between interrelated subparts, such as the simulation manikin, and the interaction and role-play in the team that creates the sense of perceived realism [22]. Our results expand on this idea, showing that increased fidelity, natural integration of different phases, and role-play as a way to promote interaction increases immersion and that these components actually might compensate for unrealistic or interruptive events or equipment during a healthcare simulation scenario.

### Limitations

The present study has some limitations. For example, it is difficult to say how the difference in time between the basic and the contextualized scenarios have affected the immersion. The ISRI score is computed using time as a denominator. In this way, the score will not be accelerated by the longer durations forced by the nature of some setups (e.g., that loading and transport has to be executed in the contextualized condition). A potential criticism of using time as a denominator is that teams can aim to increase their score by executing operations faster. This is however not an issue as the ISRI score is not used to evaluate the performance of teams. In the presented study, teams were furthermore not aware of any details of the ISRI evaluation.

Although the ISRI score is a normalized metric, differences in duration is still an important factor and further work is needed to better understand how it affects the comparison of conditions and practicalities of training sessions.

The present experiment was not preceded by a power calculation. Since ISRI is a newly developed instrument, a reliable power calculation is difficult. We hope that the results from the study can be used for power calculations to future studies including the ISRI instrument.

The presented study reveals a tendency that the immersion difference may be affected by the order of conditions. This effect is not significant and does not invalidate the main result of the study, but more studies are needed to better understand if and why it appears.

The variables manipulated between the conditions are numerous (e.g., medical scenario/clinical condition, home environment, physical, psychological, and environmental fidelity factors) and thus makes it difficult to isolate the relative impact of specific manipulations or factors on immersion.

It is also impossible to estimate how other factors outside the simulation scenarios affect immersion, as for example, individual differences, earlier experience of simulation, and expectations. The present study does not evaluate how immersion affects learning or performance. More research is needed of the appropriate level of immersion in connection to different learning goals.

## Conclusions

This study addresses how immersion in simulated prehospital training scenarios is affected by contextualization. We have studied this by applying the ISRI tool, which allows us to observe and objectively measure immersion. We conclude that contextualization of training scenarios has a positive effect on participants’ immersion experience, that it contributes to a better workflow, and promotes realistic interactions and task executions, compared to a basic simulation scenario. This suggests that efforts put into increasing physical resemblance and functional task alignment affects immersion positively. Future studies are however needed to further explore how immersion is affected by specific fidelity components (e.g., noise, home environment, or equipment props) and, perhaps more urgently, structured evaluations of the impact of immersion on learning and performance in healthcare simulations.
